# Role of Protein Quality Control Failure in Alcoholic Hepatitis Pathogenesis

**DOI:** 10.3390/biom7010011

**Published:** 2017-02-08

**Authors:** Samuel W. French, Maryam Masouminia, Sara Samadzadeh, Brittany C. Tillman, Alejandro Mendoza, Barbara A. French

**Affiliations:** 1 Harbor-UCLA Medical Center, Department of Pathology, Torrance, CA 90509, USA; maryammasouminia@dhs.lacounty.gov (M.M.); alejandromendoza@dhs.lacounty.gov (A.M.); 2 LA BioMed Research Institute, Torrance, CA 90502, USA; Sara.smdz88@gmail.com (S.S.); brittanycaratillman@yahoo.com (B.C.T.); bfrench@labiomed.org (B.A.F.)

**Keywords:** protein quality control, FATylation, ufmylation, Mallory–Denk bodies, metacaspase 1, autophagocytosis, ER stress, PERK, CHOP

## Abstract

The mechanisms of protein quality control in hepatocytes in cases of alcoholic hepatitis (AH) including ufmylation, FAT10ylation, metacaspase 1 (Mca1), ERAD (endoplasmic reticulum-associated degradation), JUNQ (juxta nuclear quality control), IPOD (insoluble protein deposit) autophagocytosis, and ER stress are reviewed. The Mallory–Denk body (MDB) formation develops in the hepatocytes in alcoholic hepatitis as a consequence of the failure of these protein quality control mechanisms to remove misfolded and damaged proteins and to prevent MDB aggresome formation within the cytoplasm of hepatocytes. The proteins involved in the quality control pathways are identified, quantitated, and visualized by immunofluorescent antibody staining of liver biopsies from patients with AH. Quantification of the proteins are achieved by measuring the fluorescent intensity using a morphometric system. Ufmylation and FAT10ylation pathways were downregulated, Mca1 pathways were upregulated, autophagocytosis was upregulated, and ER stress PERK (protein kinase RNA-like endoplasmic reticulum kinase) and CHOP (CCAAT/enhancer-binding protein homologous protein) mechanisms were upregulated. In conclusion: Despite the upregulation of several pathways of protein quality control, aggresomes (MDBs) still formed in the hepatocytes in AH. The pathogenesis of AH is due to the failure of protein quality control, which causes balloon-cell change with MDB formation and ER stress.

## 1. Introduction

Alcoholic hepatitis is characterized by the formation of the Mallory–Denk body (MDB). The formation of MDBs is an indication that the mechanisms of protein quality control have failed in the hepatocytes that have formed MDBs. The MDB is the result of proteins aggregating and accumulating in the cytoplasm of hepatocytes. These proteins failed to be turned over in a timely manner by the various mechanisms of protein degradation available to the hepatocyte. To understand how the hepatocyte turns over proteins, we investigated the various pathways involved in the failure to degrade and remove proteins by hepatocytes in human livers from patients suffering from alcoholic hepatitis.

## 2. Role of the Ufmylation and FAT10ylation Pathways to Protein Degradation by the 26S Proteasome

All investigations were carried out following the Declaration of Helsinki of 1975 and were approved by the Human Subjects Committee John F. Wolf, M.D. on 18 May 2016 (Project Identification Code: 20585).

Using formalin-fixed paraffin-embedded (FFPE) human liver biopsies from patients with alcoholic hepatitis and mice livers forming MDBs in response to DDC (5-diethoxycarbonyl-1,4-dihydrocollidine) refeeding, a marked decrease in expression of key components of the FAT10ylation and ufmylation pathways to 26S proteasome protein degradation, in proportion to the degree of MDB formation, was found [1]. The FAT10ylation pathway components that were markedly downregulated were Uba6 (ubiquitin-like modifier activating enzyme 6) and ubiquitin conjugating enzyme 1 (USE1). For the ufmylation pathway, Uba5 and ubiquitin-fold modifier conjugating enzyme 1 (Ufc1) were markedly downregulated [1]. The same components were also downregulated in the livers of mice refed DDC for seven days to induce MDB formation. There was an increase in expression of FAT10 (human leukocyte antigen F-associated transcript 10) [2,3]. MDB formation may be the result of the reduced activity of the FAT10ylation pathway of protein degradation, causing the proteins to accumulate and form aggresomes (MDBS). The FATylation pathway of protein degradation may be essential for MDB formation, since *FAT10* knockout (KO) mice refed DDC failed to form MDBs compared with wild-type mice refed DDC where numerous MDBs were formed [4].

Betaine dietary feeding given to 7-day DDC-refed mice, prevented the downregulation of Ufml, Uba5, and Uf5P1 and prevented the increased expression of FAT10 and the immunoproteasome catalytic subunit LMP7 caused by DDC refeeding [5]. A 2% betaine diet had previously been shown to largely prevent the MDB formation caused by DDC refeeding in mice [6].

This was at least partially achieved by inducing epigenetic changes in alcoholic hepatitis patients [5]. The DNA methylation levels of *Ufm1*, *Ufc1*, and *UfSP1* in the promoter CpG region were increased in the livers of alcoholic hepatitis patients. *DNMT1* and *DNMT3B* (DNA-(cytosine-5) methyltransferase 1 and 3B) mRNA levels were markedly upregulated in the alcoholic hepatitis patients. These findings support the concept that DNA methylation is the mechanism for the silencing of the Ufm1 pathway of protein quality control—which is downregulated in alcoholic hepatitis—with MDB formation as a consequence, and that this process can be prevented by the methyl-donor betaine. *S*-adenosylmethionine (SAMe) feeding, another methyl donor, also prevents MDB formation in DDC-refed mice [7].

## 3. Changes in the Activity of Metacaspase 1 and Chaperones Involved in Protein Quality Control in Alcoholic Hepatitis

Chaperones play a role in removing misfolded and aggregated proteins, compensating for the failure of the 26S proteasome to provide adequate protein quality control in hepatocytes in alcoholic hepatitis [8]. The process involves recognition of misfolded and aggregated proteins by chaperones and E3 ligases for ubiquitination and subsequent degradation through various mechanisms. For example, metacaspase 1 (Mca1), with the aid of heat shock protein 104 (Hsp 104), counteracts the aggregation and accumulation of misfolded proteins [9]. Nucleoporin p62 is involved in linking polyubiquitinated protein aggregates to the autophagy machinery [10]. The Hsp70/Hsp 40 chaperone system also plays an essential role in cell autophagy. Ydj1 is required for ubiquitin-dependent degradation of certain abnormal proteins. Also, it interacts with Ssa-1 and facilitates endoplasmic reticulum-associated degradation (ERAD) [11]. CP/97 cooperates with diverse partner proteins to help process ubiquitin-labeled misfolded proteins for recycling degradation by the 26S proteasome [12]. However, if such protein degradation mechanisms are unavailable, protection of the cellular environment from misfolded proteins is accomplished by its sequestration into two distinct inclusion bodies [13], the JUNQ (juxta nuclear quality control compartment) and the IPOD (insoluble protein deposit). We measured each of these proteins in the livers from patients with alcoholic hepatitis and from controls, by measuring fluorescent intensity morphometrically after staining the livers with fluorescent antibodies. In the alcoholic hepatitis livers fixed in formalin and embedded in paraffin (FFPE), Mca1, Hsp 104, Hsp 40, Ydj1, Ssa-1, VCP/97, and p62 were all significantly increased [8]. Mca1, Ssa-1, Hsp104, Ydj1, and VCP/97 positivity support the upregulation of the ERAD/26S proteasome pathways of protein degradation [8]. HSP40, Ssa-1, VCP/p97, and p62 positivity support the upregulation of the autophagy pathway of protein degradation [8]. Mca1, Hsp 104, and p62 positivity support the upregulation of IPOD [8]. Mca1, Ydj1, Ssa-1, Hsp104, and p62 positivity supports the upregulation of JUNQ and IPOD pathways of protein degradation [8]. These results indicate that all four of the pathways studied were upregulated to help compensate for the downregulation of ufmylation and FAT10ylation activity and the reduced autophagy due to the inhibition by upregulation of mTOR (mammalian target of rapamycin) in the alcoholic hepatitis livers assayed. This is based on the fact that the hepatocytes continue to form aggresomes (MDBs) in the alcoholic hepatitis livers.

## 4. Components of Autophagocytosis are Upregulated in Alcoholic Hepatitis

Autophagy is one of the essential pathways that maintain cellular functions by providing protein quality control via removal of misfolded and otherwise damaged proteins and providing a compartment for lysosomal enzyme digestion [14]. Autophagy-related proteins (ATG) 1–10 are the core proteins in autophagosome formation [15] that have four subgroups: a) ATG1/ULK1 complex; b) ATG6/Beclin complex that regulates the formation of the phagosome; c) ubiquitin-like complexes (LC3 and ATG 5–12) regulating vesicle expansion; d) ATG9 that is required for delivery of membranes that form the autophagosome. Accumulation of proteins in prolonged ER stress leads to intrahepatic protein aggregate formation such as MDBs. ATP and energy level reduction in the cell leads to an increase in AMP-activated protein kinase (AMPK)-related autophagy by removing the inhibitory brakes on TORC1 or activating ULK1 directly [16]. Phosphorylation of AMPK inactivates TORC 1 and activates ATG1 of the ULK1 family [17].

**Figure 1:**
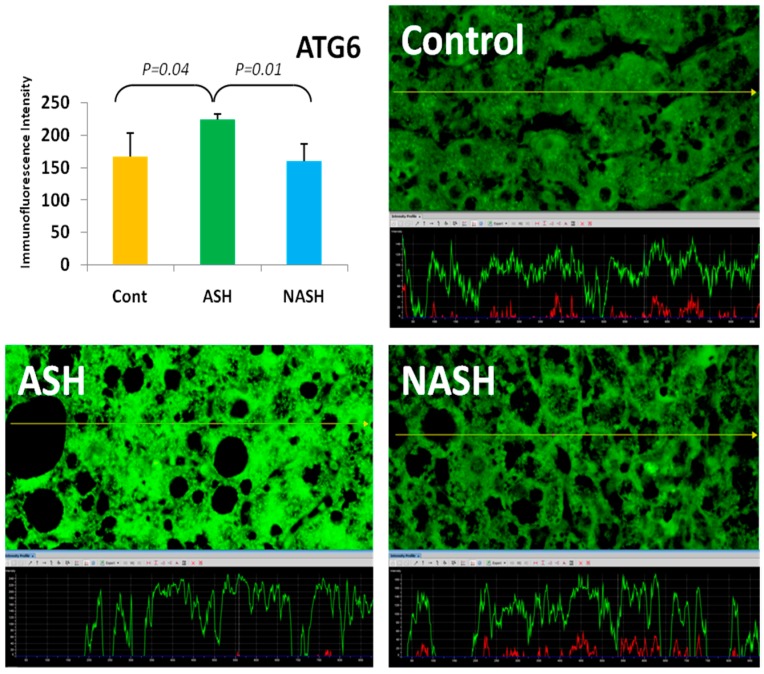
The expression of autophagy-related protein 6 (ATG6) in alcoholic steatohepatitis (ASH) was significantly upregulated compared with non-alcoholic steatohepatitis (NASH) and controls (*p* < 0.05). Its expression in NASH was slightly decreased in comparison to controls. Previously published in [21].

**Figure 2:**
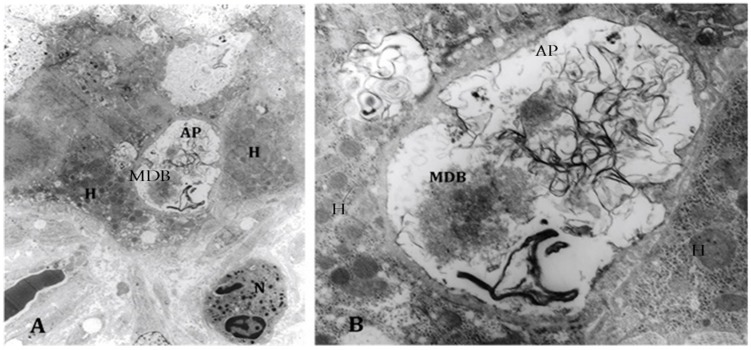
(**A**) Electron microscopy of an alcoholic steatohepatitis specimen showing an autophagosome (AP) adjacent to a neutrophil (N) and hepatocytes (H) (1458x); (**B**) higher magnification of the autophagosome shows a Mallory–Denk body (MDB) within it, mixed in with tangles of membranes (6250×). Previously published in [21].

The ATG/ULK1 complex, with the support of other proteins, translocates to the autophagosome sites [18], regulates Beclin/ATG6 and translocates ATG9 from the Golgi as an additional membrane donor for autophagosome formation. ATG4 and ATG5 are other ATG proteins interacting with the LC3 complex in autophagocytes [19]. mTOR was assayed because mTOR inhibits autophagy [20]. Also, AMPK1, ATG1, ATG4–6, and ATR were quantitated in livers from patients with alcoholic hepatitis fixed in formalin and embedded in paraffin using fluorescent antibodies and measuring the fluorescent intensity morphometrically. AMPK1 was significantly increased, as was ATG1 and ATG6 (Figure 1). ATG9, ATG5, and ATG4 were not significantly different from controls, whereas mTOR was significantly increased compared with controls. The results indicate that only some of the autophagy-dependent genes were increased. However, electron microscopic examination of the liver biopsies from patients with alcoholic hepatitis showed prominent autophagy. MDBs were present in the autophagosomes (Figure 2) seen for the first time in human liver biopsies [21].

## 5. ER Stress Response

**Figure 3:**
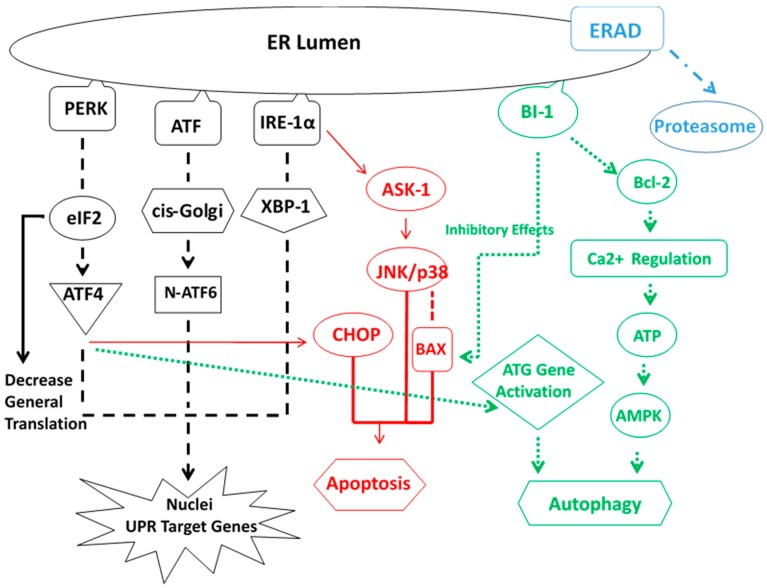
Cross-reaction of cycloprotective pathways involved in protein quality control activated by ER stress. ERAD: endoplasmic reticulum associated degradation.

“It is apparent that a complex interplay exists between the ER stress response, conditions that promote it, and those that result from it. A vicious cycle in which ER stress promotes inflammation, cell injury and steatosis and in which steatogenesis, inflammation and cell injury aggravate ER stress seems to be at play" [22]. To show the various pathways that interact in the severe ER stress reaction we offer the Figure 3 from Figure 1 in [23].

**Figure 4:**
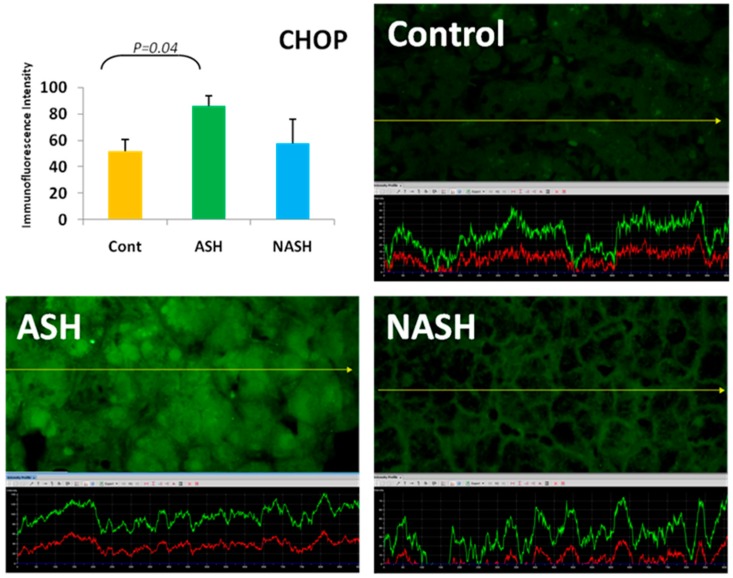
The expression of CHOP (CCAAT/enhancer-binding protein homologous protein) in ASH was significantly increased in ASH compared with controls (*p* < 0.05) and was increased compared to NASH. Its expression in NASH was increased in comparison to controls. Previously published in [21].

“ER plays an essential role in the secretory pathways and it is the destination where proteins are distributed into the endo and exocytotic pathways. Assembly, folding and disulfidation of proteins occur in the ER before exposure to the extra ER space. The concentration of proteins in the human ER is extremely high” [24] and about 13 million secretary proteins are formed per minute in hepatocytes [25]. To measure the concentration of key proteins in hepatocytes involved in ER stress in alcoholic hepatitis livers, we quantitated the proteins immunohistochemically by measuring the fluorescent intensity using morphometrics. PERK (protein kinase RNA-like endoplasmic reticulum kinase) is the first protein that increases in ER stress [26] and that phosphorylates eLF2α. This decreases the rate of mRNA translation in protein synthesis and activates ATF4 [27]. We found that PERK was significantly upregulated in alcoholic hepatitis [23]. CHOP (CCAAT/enhancer-binding protein homologous protein) was also upregulated in alcoholic hepatitis (Figure 4) [21]. CHOP colocalized with ubiquitin within MDBs [21].

IRE1α (inositol-requiring enzyme 1 alpha) is activated by p38 mitogen-activated protein kinase (MAPK), which phosphorylates and activates transcription factor CHOP. CHOP increases the expression of genes, which facilitate apoptosis [28,29]. CHOP is a multifunctional transcription factor in the ER stress response, including the activation of the inflammasome and interleukin (IL)-Iβ [30]. We previously reported that the inflammasome is activated in alcoholic hepatitis-positive livers [31]. CHOP expression also leads to cell cycle arrest. Five other factors (i.e., ATM, p21, p27, p15, and TGFβ) that inhibit liver regeneration were upregulated in livers from patients with alcoholic hepatitis [32]. Table 1 summarizes the changes in protein expression and the gain or loss of function in alcoholic hepatitis. Further studies are indicated to determine the impact of each pathway involved in protein quality control on alcoholic hepatitis balloon-cell change and MDB formation.

## 6. Conclusions

The various mechanisms involved in the liver cell protein quality control are reviewed in reference to the changes in liver cell protein turnover in human alcoholic hepatitis. The inhibition of the protein degradation mechanism observed included the FAT10ylation and ufmylation, ER stress, PERK, and CHOP upregulation and mTOR inhibition of autophagocytosis balanced against upregulation of ER stress-related ERAD autophagocytosis, metacaspase 1, JUNQ, and IPOD. Since MDBs continue to form in hepatocytes in alcoholic hepatitis where MDBs form as aggresomes of undigested malformed proteins, the overall function of the protein quality control mechanisms favor aggresome formation where alcoholic hepatitis results.

## Figures and Tables

**Table 1:** Proteins changed which contribute to loss of protein quality control in ah.

Protein	Function	Reference
Uba6	FAT10ylation pathway	[1]
USE1	FAT10ylation pathway	[1]
Ufa1	Ufmylation pathway	[1]
Uba5	Ufmylation pathway	[1]
FAT10	FAT10ylation pathway	[2,3]
Ubiquitin	26S proteasome pathway	[4]
LMP7	Immunoproteasome pathway	[5]
DNMT1	DNA methylation	[7]
DNMT3B	DNA methylation	[7]
Mca1	Chaperones pathway	[9]
Hsp104	Autophagia pathway	[9]
p62	Autophagia pathway	[10]
Hsp70/Hsp4	Autophagia pathway	[10]
Ydjl/SSa1	ERAD pathway	[11]
VCP/97	26S proteasome pathway	[12]
JUNQ	Protein sequestration	[13]
IPOD	Protein sequestration	[13]
ATG 1-10	Autophagy pathway	[15]
TORC1	Inhibits autophagy	[16]
ULK1	Inhibits autophagy	[16]
AMPK	Activates autophagia	[16]
mTOR	Inhibits autophagia	[20]
PERK	Inhibits ER stress	[26]
CHOP IL-1B	Apoptosis, inflammasome, cell cycle arrest	[31]
ATM	Inhibit liver regeneration	[32]
p21	Inhibit liver regeneration	[32]
p27	Inhibit liver regeneration	[32]
p15	Inhibit liver regeneration	[32]
TGFβ	Inhibit liver regeneration	[32]
